# Clozapine use in drug induced psychosis in Parkinson´s disease: a case report and review of literature.

**DOI:** 10.1192/j.eurpsy.2023.2208

**Published:** 2023-07-19

**Authors:** A. Sanz Giancola, P. Setién Preciados, E. Arroyo Sánchez, I. Romero Gerecther, M. Martín Velasco, C. Díaz Mayoral

**Affiliations:** Psychiatry, Hospital Universitario Príncipe de Asturias, Alcalá de Henares, Spain

## Abstract

**Introduction:**

The occurrence of psychotic symptoms induced by dopaminergic drugs marks a new phase in the course of Parkinson’s disease (PD). The term drug induced psychosis may be used when other significant psychiatric diseases are excluded in patients with no history of psychosis. The prevalence of dopaminomimetic psychosis varies from 5% to 20%. Therefore, knowledge of the psychopharmacological management of this condition is essential.

**Objectives:**

The purpose of this case report and literature review is to to learn the psychopharmacological management of this not uncommon medical complication.

**Methods:**

Descriptive case study and review of literature

**Results:**

We present the case of a 71-year-old man with a medical history of Parkinson’s disease with partial response to treatment with high doses of levodopa and carbidopa.

He was brought to the emergency department by his family due to the presence of behavioural alterations at home.

The patient reported seeing men in foam trying to harm his family. In a disjointed way in his speech, he links this idea with the delusional belief that he is being watched by electronic devices placed throughout the house. In a variegated manner he links this with a coelotypical type of discourse, however the delusional ideation remains unstructured throughout.

With no previous personal or family history of mental health and ruling out underlying organic conditions, a diagnosis of psychosis secondary to pharmacological treatment for Parkinson’s disease is presumed.

Considering the risks and benefits, it was decided to maintain the anti-Parkinson’s dose in order to avoid worsening the patient’s motor function. Therefore, after reviewing the literature, the best option was to introduce clozapine at low doses, up to 50 mg at night, with the respective analytical control. After a week’s admission, the patient began to improve psychopathologically, achieving an ad integrum resolution of the psychotic symptoms.

**Conclusions:**

Despite the availability of other antipsychotic treatments such as quetiapine or the more recent pimavanserin, clozapine remains the treatment of choice for drug-induced psychosis in Parkinson’s disease.

**Disclosure of Interest:**

None Declared

